# Global Analysis of Small Molecule Binding to Related Protein Targets

**DOI:** 10.1371/journal.pcbi.1002333

**Published:** 2012-01-12

**Authors:** Felix A. Kruger, John P. Overington

**Affiliations:** European Bioinformatics Institute, Hinxton, United Kingdom; National University of Singapore, Singapore

## Abstract

We report on the integration of pharmacological data and homology information for a large scale analysis of small molecule binding to related targets. Differences in small molecule binding have been assessed for curated pairs of human to rat orthologs and also for recently diverged human paralogs. Our analysis shows that in general, small molecule binding is conserved for pairs of human to rat orthologs. Using statistical tests, we identified a small number of cases where small molecule binding is different between human and rat, some of which had previously been reported in the literature. Knowledge of species specific pharmacology can be advantageous for drug discovery, where rats are frequently used as a model system. For human paralogs, we demonstrate a global correlation between sequence identity and the binding of small molecules with equivalent affinity. Our findings provide an initial general model relating small molecule binding and sequence divergence, containing the foundations for a general model to anticipate and predict within-target-family selectivity.

## Author Summary

Many drugs are small molecules that specifically bind to proteins involved in disease related processes. In this way, drugs modulate the function of a targeted protein and ultimately the process causing the disease. The development of drugs crucially relies on assays that measure the potency of the effect a small molecule exerts on its protein target. We compared the potencies of small molecules measured for human proteins and the corresponding (orthologous) protein in rat. Our results suggest that, after subtraction of statistical noise, most human proteins are equally susceptible to small molecule binding as their orthologs in rats. However, we identified a small number of exceptions to this rule, for example the histamine H3 receptor, a protein of the central nervous system. We also compared the potency of small molecules measured against a human protein and another member of the same protein family. In drug development it is often desired to target a protein selectively over other related proteins. The observed differences were generally greater than the statistical noise, indicating that most of the small molecules in our study have some degree of selectivity within protein families.

## Introduction

The development of medicinal chemistry lead structures into clinical candidates requires iterative testing in a variety of assays systems and frequently across different mammalian species [1]. In drug discovery, early screens are often performed with recombinant proteins, or human cell-lines heterologously expressing the desired target; later, candidate compounds are typically evaluated *in vivo* in rats and other species for efficacy and safety pharmacology. Entrance into the clinical stages of drug development then requires a switch to tests in human patients. Understanding the behavior when switching animal model species, for both the desired target mechanism, selectivity, and also for ADMET factors is crucially important. Clearly a more successful drug discovery program will have translatable pharmacology across mammalian taxa - we call this property pharmacological robustness. Pharmacological robustness between different species is limited by differences in the target protein sequence, absorption, drug metabolism and the mode of drug action, which may not be conserved between species or result in different endpoints at a phenotypic [2] level. The underlying process of molecular evolution adds stochastic noise to this transferability of function - neutral drift between orthologs and selective pressures in the evolution of functionally differentiated paralogs [3] create an ensemble of differing binding sites between and within organisms. Within organisms, the selectivity of a compound is determined by its preferential binding to one member of a protein family over other paralogs in that family. Since the process of drug discovery is often organized conceptually around pharmacologically successful gene families (such as nuclear receptors, rhodopsin-like GPCRs, various ion-channel families, and most recently, protein kinases) [4], [5], leveraging known data to develop novel selective chemotypes is of fundamental importance. Hence, understanding small molecule binding in the context of orthologous and paralogous relationships is an essential component for the systematic categorizations of both the ligand and target binding space - this field is typically now known by the term chemogenomics [6]. Chemogenomic studies have previously established a classification of human G-protein-coupled receptors (GPCRs) based on the chemical similarity of their ligands [7]–[9]. The specificity of kinase inhibitors has been evaluated within and across families of protein kinases [10]–[13] and case studies exist that examine the interplay of evolutionary relationship and binding affinity e.g. for cytochrome P450 [14] or the highly promiscuous kinase inhibitor staurosporine [15]. This compound owes its large spectrum of susceptible kinases to its interaction with the fundamentally invariant peptide bond backbone of the active site rather than individual residues therein.

The amount of publicly available small molecule bioactivity data is increasing and semantically useful annotations of these data are improving [16], [17]. For the first time it is possible to attempt the use of data integration techniques [18]–[20] for the study of ligand binding at a global level among various protein families and across species. The global perspective is challenging, the data available for this type of analysis is heterogeneous and biased for certain target classes, most prominently GPCRs and kinases. Here we report on the integration of literature inhibition and related data (Ki, IC50, EC50) measured against more than one hundred and fifty different human proteins and their rat orthologs, obtained from the ChEMBL data base. Differences in bioactivity were examined in relation to different types of evolutionary relationship (orthology and paralogy) and by comparing protein sequences on three distinct levels, sequence, known/presumed ligand-binding domain and binding site (where known). This comparison is of special importance for cases where selectivity within a gene family is required, and where selectivity needs to be preserved across model organisms (specifically rat-to-human).

## Results

### Robustness of small molecule binding across species

**Figure 1:**
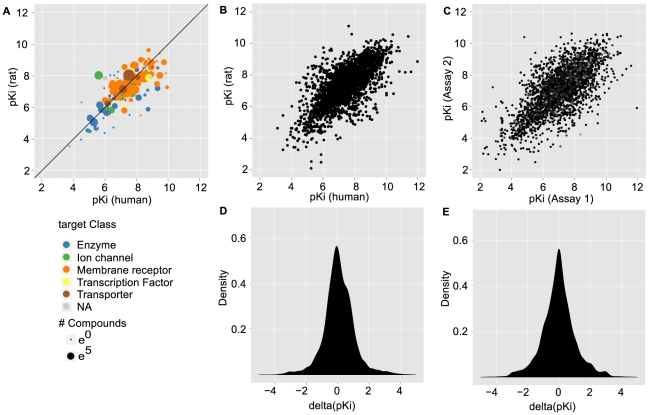
Differences in small molecule binding for human to rat orthologs. (a) Summary scatter plot. Positions along axes report the median binding affinity of all compounds against the human target (x-axis) and its ortholog in rat. Points are scaled according to the number of different compounds tested against a pair of orthologs. (b) Scatter plot of individual compound binding affinities for the human targets (x-axis) and their orthologs in rat (y-axis). (c) Scatter plot of the affinities observed when comparing results from different assays of the same compound and target (1500 human targets and 1500 rat targets). (d) Distribution of differences in binding affinity between the human target and its ortholog in rat. Positive values indicate higher affinity for the human target and vice versa. (e) Distribution of the differences observed when comparing binding affinities for the same compound and target in different assays.

As a first step to estimate the robustness of small molecule binding globally, we compared bioactivity data for compounds that have been tested against a human target and its rat ortholog. In principle, comparison with other species is possible but in practice is limited by the availability of data. The data bias towards rat reflects the ubiquitous use of rat as an *in vivo* model organism in pharmacology studies [21]. From the ChEMBL database (version 10 [22]), we retrieved 2,782 instances of compounds tested against both, the human target and its ortholog in rat. The human-to-rat data is made up of 151 different orthologous pairs. The observed bioactivities expressed as log transformed dissociation constants pKi range from 4 to 12 (i.e. across a broad range from high 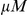 to single digit 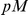). For our purposes, the data is also rich across the concentration range known to be important for acceptable clinically efficacious modulation of a target [23]. We calculated the difference in bioactivity for each pair of orthologs and a given ligand. The density distribution of differences in bioactivity has a central peak at 0 and is non-normal as established by Anderson-Darling test [24] (p<2e-16). This is in support of the trivial model that small molecules bind, on average, a human target and its orthologs in other species with the same affinity. An analysis using Pearson's correlation coefficient indicates a highly significant linear relationship between bioactivities measured against human and rat targets (r = 0.71, p<2e-16). As a measure of both publication and abstraction errors within the ChEMBL database and also for between-lab variability, and to estimate the deviations introduced by inter-assay comparison of binding data, we calculated the differences in bioactivity (expressed in terms of a log pKi) between assays that measured the effect of identical ligands on identical targets. To ensure the resulting distribution was comparable to the distribution of differences in activity between human-to-rat orthologs, this distribution was constructed as a composite of 1.500 randomly picked inter-assay differences for human targets and 1.500 randomly picked inter-assay differences for rat targets. Thus, the number of values available for human-to-rat orthologs (2.782) was approximately matched. For targets having more than two assays, group averages were taken before calculating the difference. The resulting distribution closely resembles the distribution for orthologs, further supporting the hypothesis that binding is globally conserved between orthologs. A summary of the data is provided in Figure 1.

To identify human proteins that have significant inter-species (here human-rat) differences in ligand binding, we carried out two-sided Mann-Whitney U tests, comparing the distribution of binding differences observed for a single pair of orthologs to the control distribution of inter-assay differences. P-values were corrected for multiple comparisons using Bonferroni adjustment. Results with significant deviation from the distribution mean (p<0.05) are reported in Table 1. To estimate the chemical diversity, ligand sets for each target were clustered using a distance matrix calculated from fingerprint similarities and numbers of clusters are reported (see Methods section Compound Clustering). This large-scale comparison of human versus rat orthologs shows that some chemotypes have a systematic preference, or bias in binding affinity, for one species over the other. For example, the significant preference of pyrrolidine-containing Histamine H3 receptor (HRH3) antagonists for the human over the rat ortholog has been previously reported in the medicinal chemistry literature [25]–[30], but here is rediscovered using our unbiased and automated analysis.

**Table 1:** Proposed pairs of human to rat orthologs with species specific pharmacology.

Name (Uniprot)	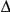	#cmpds	#chemotype	mapped Pfam
Histamine H3 receptor	0.59	325	30	7tm_1
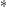 Neuronal ACh-receptor 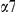	−2.33	49	3	Neur_chan_LBD
Serotonin transporter	−0.42	309	27	SNF
Fructose-1,6-bisphosphatase	0.82	50	2	FBPase
NaV type X 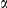 -subunit	0.77	45	3	Ion_trans
Gonadotropin-releasing hormone receptor	0.54	118	10	7tm_1
D-amino-acid oxidase	1.68	14	2	DAO
Neurokinin 1 receptor	1.74	20	2	7tm_1
Adenosine A1 receptor	−0.51	78	16	7tm_1
Adenosine A2a receptor	0.41	112	15	7tm_1
Androgen Receptor	0.90	25	3	Hormone_recep
Cathepsin S	2.02	9	1	Peptidase_C1
Glucagon receptor	1.26	12	2	not mapped
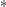 Urotensin II receptor	−0.49	56	4	7tm_1

Reported are target pairs where observed differences in binding affinity deviate significantly from the control distribution. 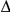 represents mean potency differences. Positive values of 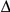 designate preferential binding to the human ortholog and negative values preferential binding to the rat ortholog. Asterisks mark values which are artifacts of faulty annotation. The number of tested compounds is reported in the column #cmpds and #chemotypes reports the number of chemotypes determined by hierarchical clustering.

Two of the reported cases are problematic because the source documents report functional *in vivo* assay data rather than emphin vitro binding data. 43 of the 56 Urotensin II receptor antagonists have been tested for their potency in a calcium-flux assay but by an error of annotation, these were labelled as *in vitro* binding data. A similar misannotation was detected for the neuronal acetylcholine receptor subunit 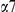. These findings were used to set curation priorities for future releases of the ChEMBL.

### Conservation of small molecule binding between paralogs

Gene duplications produce two copies of the original gene within one genome [31]. Duplicated genes, or paralogs, experience less selective pressure and typically one gene copy can develop new divergent functions (if subsequently fixed in the population). To examine the conservation of small molecule binding among paralogs, we identified pairs of human paralogs using a pre-calculated phylogenetic tree from Ensembl Compara [32] and small molecule binding data was retrieved from the ChEMBL. Activities of 20,309 compounds against 516 different human targets were obtained and 651 pairs of human paralogs identified, and differences in binding affinity were calculated for a total of 41,733 combinations. These calculated differences were randomly assigned with a positive or negative prefix (in order to allow comparison with the data for the orthologous pairs). Thus, we introduced an artificial binomial grouping of measured differences, imitating the grouping by species for orthologs, and facilitating the comparison of both sets. In contrast to orthologs, observed differences in binding affinity were larger, and the proportion of homologous pairs binding targets with the same affinity smaller. A comparison of orthologs and paralogs is presented in the next section.

### Evolutionary relationship and small molecule binding

**Figure 2:**
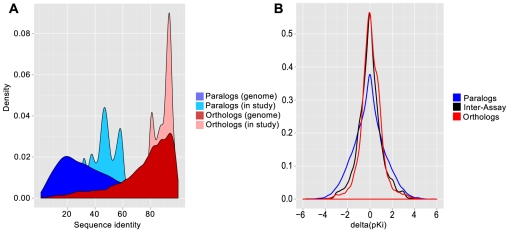
(a) Distributions of pairwise sequence identity between orthologs (red) and paralogs (blue). Distributions in the front represent all pairs retrieved from Ensembl Compara and distributions in the back pairs of targets from our analysis. (b) Distributions of differences in binding affinity between the human target and its rat ortholog (red curve) and distribution of differences in binding affinity between human paralogs (blue curve). For comparison, the distribution of inter-assay differences is outlined (black curve).

Paralogs arise from gene duplication events within a species and, owing to their redundancy, often evolve to develop divergent functions. The independent evolution of orthologs on the other hand is the consequence of a speciation event [33]. Compared to paralogs, mammalian orthologs have diverged relatively recently [34]. This is reflected in the distribution of pair-wise sequence identities observed between human paralogs and human-rat orthologs (Figure 2). Most frequently, the sequence identity between human paralogs in Ensembl Compara [32] is approximately 30% while human to rat orthologs are most frequently observed with sequence identity of around 90%, as shown in Figure 2. Of note, the distributions of pair-wise sequence identity for targets in our analysis deviate from the genome-wide distribution. Paralogs in this analysis set generally have higher sequence identity compared to all human paralogs. This can be explained by the bias from the rational identification and anticipation of selectivity issues within a gene family, where scientists are typically concerned about the most closely related sequences for two reasons, overlapping signaling and presumed higher likelihood of cross-reactivity of ligands. Within our data, this is especially clear for the rhodopsin-like GPCR and kinase families. Comparing differences in binding affinity between pairs of paralogs and orthologs, we observed a higher conservation of the binding affinity for given ligands between pairs of orthologs compared to pairs of paralogs. An overlay of the distributions of differences in binding affinity for both, human to rat orthologs and human paralogs is shown in figure 2. Both distributions can be approximately described by a Laplace distribution. Notably, the scale parameter b, which describes the spread of the distribution, is b = 0.7 for the paralogs and b = 1.3 for human-to-rat orthologs (as expected by the differing selective pressures within these two sets). The variance of measured binding affinities is significantly different between orthologs and paralogs as established by Levene's test [35] (p<2.2e-16). Hence, pairs of paralogs are less likely to bind ligands with the same affinity and the observed differences in binding affinity are larger compared to human to rat orthologs.

### Sequence identity and conservation of binding

The observation that small molecule binding is less conserved in paralogs compared to orthologs (and the distinct behavior of these two sets) led us to enquire for the molecular mechanism by which paralogs acquire different binding affinities for one ligand. It is established that the vast majority of differences between pairs of proteins have minimal functional effect [36] and it would be expected that the same applies to the binding of small molecules. To investigate this, we compared the entire sequences of paralogs. Furthermore we compared sequences of the presumed structural domain containing the ligand-binding site. The algorithm used to map binding sites to domains is described in Methods - Mapping binding sites to Pfam domains and a summary selection of the 25 most frequent domains which account for more than 50% of all observations in the ChEMBL target dictionary is shown in Table 2. Results of the mapping are discussed in Text S1 (see also Figures S1, S2). For two classes of targets, GPCRs and kinases, we also compared targets based on alignments of the known/presumed binding site. An analysis on each level showed a highly significant but weak correlation between simple sequence identity and the absolute difference in bioactivity on all levels of the comparison. It is worth noting that the strength of the correlation increases with the level of precision of the sequence comparison. Spearmans non-parametric correlation coefficients were −0.082 (p<2.2e-16) on the level of the full sequence, −0.10 (p<2.2e-16) on the level of the domain predicted to contain the binding site and −0.21 (p<2.2e-16) on the level of the binding site. We carried out the same analysis for human-to-rat orthologs and did not detect a significant correlation of whole protein sequence identity and differences in small molecule binding (p = 0.34). This could be due to a smaller sample size (2,782 for orthologs versus 41,733 for paralogs) but more likely reflects the contrast between the functional conservation of orthologs and different degrees of diversification for paralogs.

**Table 2:** The most frequent ligand binding domains in the ChEMBL target dictionary.

Pfam accession	#predicted	% of all predicted
7tm_1	502	12.48
Pkinase	404	10.05
Pkinase_Tyr	146	3.63
Neur_chan_LBD	139	3.46
Ion_trans	114	2.84
Trypsin	93	2.31
Hormone_recep	82	2.04
p450	71	1.77
ANF_receptor	71	1.77
PDEase_I	49	1.22
Beta-lactamase	39	0.97
Carb_anhydrase	36	0.90
Peptidase_C1	35	0.87
Guanylate_cyc	34	0.85
HATPase_c	34	0.85
Hist_deacetyl	33	0.82
SNF	33	0.82
adh_short	33	0.82
Asp	31	0.77
Y_phosphatase	29	0.72
Peptidase_M10	28	0.70
Tubulin	28	0.70
DHFR_1	28	0.70
Phospholip_A2_1	26	0.65
Metallophos	24	0.60

The Pfam accessions of the 25 most frequent binding site containing domains are listed together with the number of times a domain occurs in the ChEMBL target dictionary (# predicted) and its proportion of all predicted binding sites (% of all predicted).

**Figure 3:**
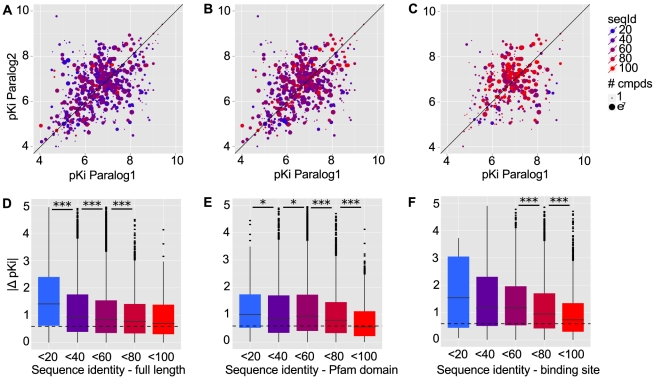
Differences in small molecule binding for human paralogs. Sequence identity was measured on three levels: (a,d) Full sequence, (b,e) sequence of the domain containing the binding site and (c,f) residues of GPCR and kinase binding sites. Scatter plots of binding affinities measured against pairs of human paralogs are shown in the top row. Points represent median affinities of all compounds measured against a given combination of targets. Box plots in the bottom row represent the distribution of measured differences in binding affinity for pairs of targets having 0–20%, 20–40%, 40–60%, 60–80% and 80–100% identity. The level of significance of differences between bins are indicated with one (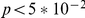), two (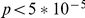) or three asterisks (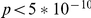). The dashed horizontal line indicates the mean inter-assay difference for human targets.

Figure 3 shows the relationship of sequence identity and difference in bioactivity. The mean difference in binding affinity for pairs of paralogs with sequence identity > = 80% is similar to the observed inter-assay difference for human targets. On the other hand, targets with sequence identity below 80% on average have activity differences that are higher than the inter-assay deviation. A prior study had shown that kinases statistically have similar SAR properties above a sequence identity threshold of 60% [37]. On the level of the binding site containing domain it appears that below a sequence identity of 80% pairs of paralogs exhibit increasingly divergent binding towards the same target, while binding is statistically conserved above this threshold. On the level of the binding site, the average difference in binding correlates strongest with sequence differences.

### The magic methyl revisited

**Figure 4:**
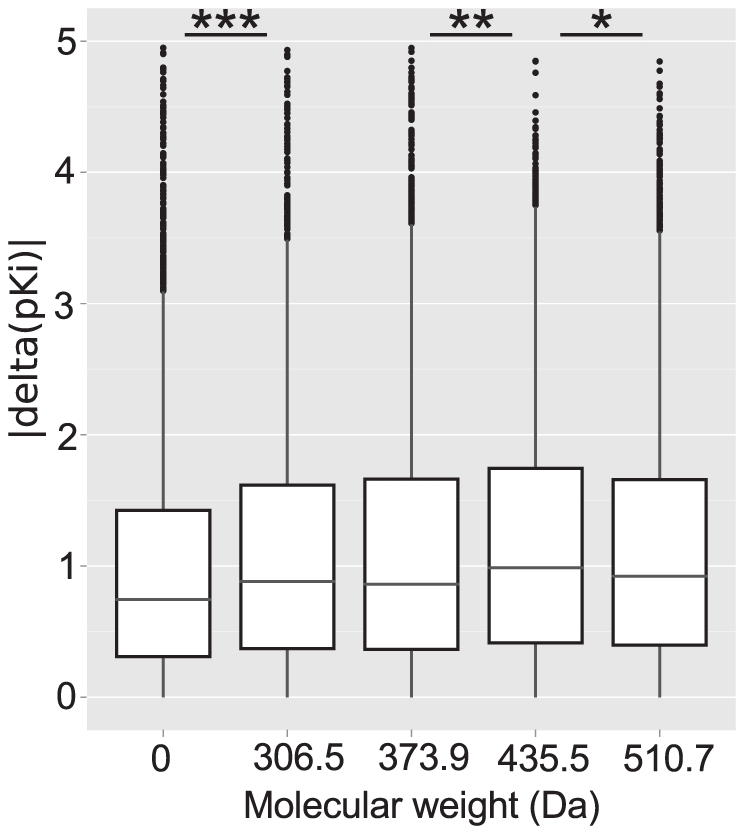
Molecular weight and absolute differences in binding affinity. Box plots show distributions of differences in binding affinity for small molecules grouped by equally sized molecular weight bins for paralogs. Each bin contains the same number of values and lower bin limits are shown below each box. Anova type multiple testing was carried out to assess the significance of differences between neighbouring groups and levels of significance are indicated with one (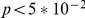), two (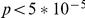) or three asterisks (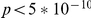).

**Figure 5:**
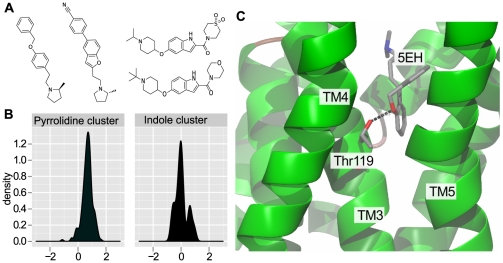
Species specific binding of small molecules for the HRH3. (a) Exemplary compounds from two clusters are shown. (b) Distributions of differences observed for compounds from the two respective clusters. Pyrrolidine containing componds bind the human target with higher affinity, while most compounds with an indole scaffold bind the human and rat HRH3 with equal affinity. (c) The substitution Thr119Ala is represented by a grey threonine side chain in the human receptor. The displayed ligand is the HRH1 antagonist Doxepin (5EH) and the nearest distance to Thr119 is 2.7A.

Medicinal chemistry experience shows that the addition of a single methyl group to the structure of a lead compound can change binding affinity by one order of magnitude [38]–[41]. Classically, this paradigm is associated with the small molecule part of a binding interaction as the ligand is amenable to chemical modification. In the light of evolutionary relationships, magic methyls - or rather - magic residues, can occur when mutations in the amino acid sequence (de)stabilize the complex of a homologous target and artificial ligand. The expectation would be that the binding of larger molecules is more likely to be affected by a mutation in or near the binding site, as molecules of greater size rely on a larger number of interactions with the target protein [42]. Following this hypothesis, we examined differences in ligand binding between paralogs in terms of molecular size (approximated here by molecular weight) of the ligand and divided all compounds in our analysis into molecular weight bins. Adaptive binning was used to obtain five groups containing equal numbers of compounds. An analysis of variance (Anova) F test (F = 15.0, p<2.8e-12) suggests that there are significant differences between the groups and multiple testing was carried out to examine the differences between individual groups (see Figure 4). Analogous to the above, the data for human to rat orthologs was binned by molecular weight of the ligand and an Anova F test (F = 5.6, p<1.6e-4) suggest that there is a significant difference between groups but sample sizes are smaller and multiple testing is less conclusive (see Figure S9). The differences observed when grouping ligands by molecular weight are in support of our magic residue hypothesis according to which larger molecules would be more likely to interact with residues in or near the binding site and thus would sample otherwise neutral mutations. In this context, it is difficult to distinguish between physiologically neutral substitutions in orthologs and non-homologous changes in paralogs, because a number of paralogs bind, like orthologs, their endogenous ligand with equal affinity (eg. muscarinic acetylcholine receptors, where different receptor subtypes exist) and thus behave like pseudo-orthologs. Our findings also implies that increasing the molecular size of a ligand can promote selectivity against targets within the same family if substitutions are present near the binding site. The correlation observed between the absolute difference in binding affinity and molecular weight is small (Spearman's Rho of only 0.062) but highly significant, suggesting that only a small subset of the pairs in our analysis have substitutions in or near the binding site. We propose that differences in small molecule binding between homologous targets arise from physiologically neutral mutations that only by chance become relevant when interacting with artificial ligands. These chances increase slightly with lower sequence identity but ultimately depend on whether an artificial ligand will sample mutations through direct or indirect interactions. As an example, the distinct differences between the rat and human ortholog of the histamine H3 receptor (HRH3) depend on the chemotype of the ligand. Using hierarchical clustering, we were able to show that pyrrolidine-containing ligands of the HRH3 create differential responses between human and rat HRH3s, whereas ligands based on an indole-core bind both targets with similar affinity (see Text S1 and Figures S5, S6). A homology model constructed from available GPCR structures suggests that a substitution of Thr119 in the human for Ala 119 in the rat receptor is within 2.7A of the modelled ligand Doxepin and is likely the cause of species-specific pharmacology of these otherwise very similar receptors (see Figures 5, S3, S4, Datasets S1, S2 and Text S1). Another target with species-specific binding affinities identified in this study is the serotonin transporter. Clustering of the associated small molecules revealed that compounds with an aminochroman-5-carboxamide core have a preference for the rat ortholog (see Figures S7, S8 and Text S1).

## Discussion

Our analysis shows that differences in ligand binding are significantly larger and more frequent between pairs of paralogs compared to pairs of orthologs. These findings are complementary to a study by Peterson that observed that, at the same level of sequence similarity, structural differences between paralogs are larger compared to orthologs [43]. We confirm, on a global scale, a working hypothesis in the field of medicinal and biological chemistry, intuitively based on the notion that orthologs fulfill the same function in two species while duplicated genes within one species are free to evolve to functional divergence. However, for the first time, we demonstrate this truism with analysis of a large-scale pharmacologically relevant data set. Consistent with our data is that the conservation of ligand binding depends to a large degree on few but crucial mutations in the binding site more than overall sequence identity. Our study demonstrates that the magic methyl paradigm applies not only to ligands but equally to the binding site of a protein, and that crucial substitutions override the underlying correlation of sequence identity and ligand binding. Our study differs from prior chemogenomic studies [44], [45] in its scope, which is global and integrates experimental results from different literature sources. The heterogeneity of the data imposes unknown challenges and requires new approaches to analyzing the data. Different measurement techniques and report formats introduce drastic deviations between single measurements. However, these smooth out from a global point of view, due to the sheer bulk of heterogeneous data. We anticipate a significant increase in the usefulness of global models as more bioactivity becomes available in parseable formats.

## Methods

### Source data and preprocessing

All data was collected from the ChEMBL database (version 10 [22]). Activities were filtered to contain only data from binding assays (assay-type: ‘B’) that could be mapped directly to a molecular target, in other words the mapping to targets was unambiguous. Permitted into the analysis were assays with results reported in either of the following units: Ki, IC50, EC50, pA2, pKi. Units were converted where necessary to be comparable to pKi values and duplicates excluded. Tables containing source data for comparisons of inter-assay data, human to rat ortholog data and paralog data are included as Tables S1, S2 and S3.

### Similarity metrics

For global sequence comparisons we used precalculated alignments from Ensembl Compara [32]. Sequence identity was determined as the fraction of residues that are identical between the overlapping regions of paralogous sequences. Binding site-containing domains were identified using a heuristic prediction algorithm described below. As an underlying domain classification system, we used Pfam [46], [47]. Domains containing the binding-site of paralogous proteins were aligned and sequence identity determined. Comparisons of binding sites were carried out for two target classes, Kinases and non-olfactory GPCRs. The positions of residues interacting with a given ligand were adopted from Surgand [7] for GPCRs and from Kinase SARfari [48] for kinases.

### Statistical analysis

All statistical analysis was carried out using the statistical analysis program R [49]. Used functions are specified in Table S4.

### Mapping binding sites to Pfam domains

Binding sites were mapped to Pfam domains using a simple, heuristic algorithm. In a first step, Pfam domain annotations were retrieved from the Pfam database. Most targets in ChEMBL contain only a single domain (Figure S1) and for those it is reasonable to assume that in a vast majority of cases the binding site is contained within that domain. A dictionary of binding-site containing domains was thus created and subsequently matched to ChEMBL targets. Binding-sites of targets matching only one domain are immediately mapped to this domain. All targets having more than one domain are matched against the domains extracted in the first step and if one of these domains is present in the target, the ligand binding site is mapped to this domain. If a target sequence matches two or more domains identified in the first step, the binding site is mapped to the domain with the highest count of occurrence among single domain targets. A discussion of the results of this mapping is provided in Text S1.

### Compound clustering

LINGO fingerprints have been previously described as a reliable fingerprint descriptor that is easy to calculate [50]. We used the OpenEye [51] software to calculate LINGO fingerprints for all ligands and calculated pairwise Tanimoto coefficients. A distance matrix was constructed and hierarchical clustering carried out using the complete linkage algorithm. Clusters were then determined using a Tanimoto cut-off of 0.75. This value was chosen to separate different chemical series. Table S5 is a record of database identifiers and cluster associations of all investigated compounds.

## Supporting Information

Dataset S1 **PDB file for the homology model of the human HRH3.** The model was constructed from template structures of the Histamine H1 receptor (3rze), as well as the dopamine D3 receptor (3pbl), the human beta 2 adrenergic (2rh1, 3d4s, 3ny8, 3nya) and the turkey beta 1 adrenergic receptor (2vt4).
(TXT)

Dataset S2 **PDB file for the homology model of the rat HRH3.** The model was constructed from template structures of the Histamine H1 receptor (3rze), as well as the dopamine D3 receptor (3pbl), the human beta 2 adrenergic (2rh1, 3d4s, 3ny8, 3nya) and the turkey beta 1 adrenergic receptor (2vt4).
(TXT)

Figure S1 **Histogram of the number of unique Pfam domains occurring in each protein in the ChEMBL target dictionary.**
(EPS)

Figure S2
**Cumulative log-log plot showing on the x-Axis the frequency of predicted binding-site containing domains among target proteins and on the y-Axis the number of domains with frequency>x.** Over a large range of values the distribution can be approximated by a straight line, which is indicative of a power-law distribution. The fitted curve corresponds to a power-law function 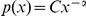 where C is the number of domains (531) and 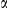 is 2.12.(EPS)

Figure S3 **Model structure of the human Histamine H3 recepor.** Thr119 (which is substituted to Ala119 in the rat ortholog) is represented by a ball and stick model of the threonine side chain in the human receptor. Displayed ligands were adopted from the template structures.
(EPS)

Figure S4 **Model structure of the human Histamine H3 receptor.** Close-up view of the THR119 residue and measured distance to one of the template ligands, Doxepin (2.69A).
(EPS)

Figure S5 **Hierachical clustering of HRH3 ligands.** Cluster 24 is by far the largest cluster representing mainly pyrrolidine-containing antagonists while cluster 10 represents imidazole-containing antagonists.
(EPS)

Figure S6 **Cluster specific distributions of differences in binding affinity.** Most of the indole based antagonists of cluster 17 bind the rat ortholog with equal affinity, while the pyrrolidine-containing antagonists (contained in cluster 24) have a marked preference for the human receptor.
(EPS)

Figure S7 **Hierachical clustering of Serotonin transporter ligands.** The two largest clusters contain 121 and 42 compounds respectively.
(EPS)

Figure S8 **(a) Cluster specific distributions of differences in binding affinity for the Serotonin transporter.** The majority of compounds in cluster 22 have ten-fold higher potency against the rat ortholog. Cluster 26 and the majority of the remaining compounds (singletons) have a slight preference for the rat ortholog. (b) 2D representation of an exemplary compound from cluster 22.
(EPS)

Figure S9
**Molecular weight and absolute differences in binding affinity.** Box plots show distributions of differences in binding affinity for small molecules grouped by equally sized molecular weight bins for orthologs. Each bin contains the same number of values and lower bin limits are shown below each box. Anova type multiple testing was carried out to assess the significance of differences between neighbouring groups and levels of significance are indicated with one (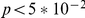), two (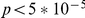) or three asterisks (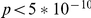). For orthologs, the only significant difference is between the group of compounds with molecular weight 375.5–422.6 Da and the group of compounds with molecular weight >483.3 Da.(EPS)

Table S1 **Inter-assay variation of measured binding affinities.** This tab-delimited table summarizes the data underlying the inter-assay analysis. The column ‘prefName’ provides the canonical name of the target, ‘tid’ the database identifier of the human ortholog, ‘Afnty1/2’ the pKi measured in each group of assays, ‘molregno’ the database identifier of the small molecule, ‘measured’ the number of measurements and ‘diff’ the difference in binding affinity.
(TXT)

Table S2 **Human-to-rat orthologs.** This tab-delimited table summarizes the data underlying the analysis of human-to-rat orthologs. The column ‘prefName’ provides the canonical name of the target, ‘Uniprot1/2’ the identifier of the human/rat ortholog, ‘Afnty1/2’ the pKi against the human/rat ortholog, ‘molregno’ the database identifier of the small molecule.
(TXT)

Table S3 **Human paralogs.** This tab-delimited table summarizes the data underlying the analysis of human paralogs. ‘Accession1/2’ give the Uniprot identifiers of the targets compared, ‘seqId’ the sequence identity between these targets, ‘molregno’ the database identifier of the small molecule, ‘Afnty1/2’ the measured affinities.
(TXT)

Table S4 **R functions.** This tab-delimited table summarizes the R functions used for statistical analysis in this article.
(TXT)

Table S5 **Clustered ligands.** This tab-delimited table lists all ligands and cluster associations for 15 protein targets with differences in small molecule binding.
(TXT)

Text S1 **This document provides additional data and analysis in support of the main text.** In the first section, results of the mapping of small molecule binding to structural domains is discussed. The second and third section describe the species specific pharmacology of the histamine H3 receptor and the serotonin transporter.
(PDF)

## References

[1] George CF (1980). Clinical pharmacology. Drug development.. Br Med J.

[2] McGary KL, Park TJ, Woods JO, Cha HJ, Wallingford JB (2010). Systematic discovery of nonobvious human disease models through orthologous phenotypes.. Proc Natl Acad Sci U S A.

[3] Kimura M (1981). Possibility of extensive neutral evolution under stabilizing selection with special reference to nonrandom usage of synonymous codons.. Proc Natl Acad Sci U S A.

[4] Hopkins AL, Groom CR (2002). The druggable genome.. Nat Rev Drug Discov.

[5] Overington JP, Al-Lazikani B, Hopkins AL (2006). How many drug targets are there?. Nat Rev Drug Discov.

[6] Caron PR, Mullican MD, Mashal RD, Wilson KP, Su MS (2001). Chemogenomic approaches to drug discovery.. Curr Opin Chem Biol.

[7] Surgand JS, Rodrigo J, Kellenberger E, Rognan D (2006). A chemogenomic analysis of the transmembrane binding cavity of human G-protein-coupled receptors.. Proteins.

[8] Gloriam DE, Foord SM, Blaney FE, Garland SL (2009). Definition of the G protein-coupled receptor transmembrane bundle binding pocket and calculation of receptor similarities for drug design.. J Med Chem.

[9] van der Horst E, Peironcely JE, Ijzerman AP, Beukers MW, Lane JR (2010). A novel chemogenomics analysis of G protein-coupled receptors (GPCRs) and their ligands: a potential strategy for receptor de-orphanization.. BMC Bioinf.

[10] Fabian MA, Biggs WH, Treiber DK, Atteridge CE, Azimioara MD (2005). A small moleculekinase interaction map for clinical kinase inhibitors.. Nat Biotechnol.

[11] Karaman MW, Herrgard S, Treiber DK, Gallant P, Atteridge CE (2008). A quantitative analysis of kinase inhibitor selectivity.. Nat Biotechnol.

[12] Metz JT, Johnson EF, Soni NB, Merta PJ, Kie L (2011). Navigating the kinome.. Nat Chem Biol.

[13] Brooijmans N, Chang YW, Mobilio D, Denny RA, Humblet C (2010). An enriched structural kinase database to enable kinome-wide structure-based analyses and drug discovery.. Protein Sci.

[14] Ekroos M, Sjögren T (2006). Structural basis for ligand promiscuity in cytochrome P450 3A4.. Proc Natl Acad Sci U S A.

[15] Tanramluk D, Schreyer A, Pitt WR, Blundell TL (2009). On the origins of enzyme inhibitor selectivity and promiscuity: a case study of protein kinase binding to staurosporine.. Chem Biol Drug Des.

[16] Overington J (2009). ChEMBL. An interview with John Overington, team leader, chemogenomics at the European Bioinformatics Institute Outstation of the European Molecular Biology Laboratory (EMBL-EBI). Interview by Wendy A. Warr.. J Comput Aided Mol Des.

[17] Taylor KR, Gledhill RJ, Essex JW, Frey JG, Harris SW (2006). Bringing Chemical Data onto the Semantic Web.. J Chem Inf Mod.

[18] Arens Y, Chee CY, Hsu CN, Knoblock CA (1993). Retrieving and integrating data from multiple information sources.. Int J Intell Coop I.

[19] Halevy A, Rajaraman A, Ordille J (2006). Data integration: the teenage years.. http://dl.acm.org/citation.cfm?id=1182635.1164130.

[20] Sujansky W (2001). Heterogeneous Database Integration in Biomedicine.. J Biomed Inf.

[21] Cozzi J, Fraichard A, Thiam K (2008). Use of genetically modified rat models for translational medicine.. Drug Discov Today.

[22] Bellis LJ, Akhtar R, Al-Lazikani B, Atkinson F, Bento AP (2011). ChEMBL 10.. ftp://ftp.ebi.ac.uk/pub/databases/chembl/ChEMBLdb/releases/chembl10/.

[23] Gleeson MP, Hersey A, Montanari D, Overington J (2011). Probing the links between in vitro potency, ADMET and physicochemical parameters.. Nat Rev Drug Discov.

[24] Stephens MS, D'Agostino RB, Stephens MS (1986). Tests for the uniform distribution.. Goodness-of-Fit Techniques.

[25] Nersesian DL, Black LA, Miller TR, Vortherms TA, Esbenshade TA (2008). In vitro SAR of pyrrolidine-containing histamine H3 receptor antagonists: trends across multiple chemical series.. Bioorg Med Chem Lett.

[26] Black LA, Nersesian DL, Sharma P, Ku YY, Bennani YL (2007). 4-[6-(2- Aminoethyl)naphthalen-2-yl]benzonitriles are potent histamine H3 receptor antagonists with high CNS penetration.. Bioorg Med Chem Lett.

[27] Esbenshade TA (2004). Pharmacological Properties of ABT-239 [4-(2- -benzofuran-5- yl)benzonitrile]: I. Potent and Selective Histamine H3 Receptor Antagonist with Drug-Like Properties.. J Pharmacol Exp Ther.

[28] Ireland-Denny L, Parihar AS, Miller TR, Kang CH, Krueger KM (2001). Species-related pharmacological heterogeneity of histamine H(3) receptors.. Eur J Pharmacol.

[29] Ligneau X, Morisset S, Tardivel-Lacombe J, Gbahou F, Ganellin CR (2000). Distinct pharmacology of rat and human histamine H(3) receptors: role of two amino acids in the third transmembrane domain.. Br J Pharmacol.

[30] Lovenberg TW, Pyati J, Chang H, Wilson SJ, Erlander MG (2000). Cloning of rat histamine H(3) receptor reveals distinct species pharmacological profiles.. J Pharmacol Exp Ther.

[31] Koonin E (2005). Orthologs, paralogs, and evolutionary genomics.. Annu Rev Genet.

[32] Vilella AJ, Severin J, Ureta-Vidal A, Heng L, Durbin R (2008). EnsemblCompara GeneTrees: Complete, duplication-aware phylogenetic trees in vertebrates.. Genome Res.

[33] Fitch WM (1970). Distinguishing Homologous from Analogous Proteins.. Syst Zool.

[34] Tatusov RL (1997). A Genomic Perspective on Protein Families.. Science.

[35] Fox J, Weisberg S (2011). An R Companion to Applied Regression. 2^nd^ edition.

[36] Bowie JU, Reidhaar-Olson JF, Lim WA, Sauer RT (1990). Deciphering the message in protein sequences: tolerance to amino acid substitutions.. Science.

[37] Vieth M, Sutherland JJ, Robertson DH, Campbell RM (2005). Kinomics: characterizing the therapeutically validated kinase space.. Drug Discov Today.

[38] Shamovsky I, Connolly S, David L, Ivanova S, Nordén B (2008). Overcoming Undesirable hERG Potency of Chemokine Receptor Antagonists Using Baseline Lipophilicity Relationships.. J Med Chem.

[39] Davis AM, Teague SJ (1999). Hydrogen bonding, hydrophobic interactions, and failure of the rigid receptor hypothesis.. Angewandt Chem Internatl Ed.

[40] Zhi L, Tegley CM, Marschke KB, Jones TK (1999). Switching androgen receptor antagonists to agonists by modifying C-ring substituents on piperidino-3,2-quinolinone.. Bioorg Med Chem Lett.

[41] Hopkins A (2004). Ligand efficiency: a useful metric for lead selection.. Drug Discov Today.

[42] Hann MM, Leach AR, Harper G (2001). Molecular Complexity and Its Impact on the Probability of Finding Leads for Drug Discovery.. J Chem Inf Mod.

[43] Peterson ME, Chen F, Saven JG, Roos DS, Babbitt PC (2009). Evolutionary constraints on structural similarity in orthologs and paralogs.. Protein Sci.

[44] van der Horst E, Peironcely JE, van Westen GJP, van den Hoven OO, Galloway WRJD (2011). Approaches for Receptor Deorphanization and Extensions of the Chemogenomics Concept to Phenotypic Space.. Cur Top Med Chem.

[45] Wichard JD, ter Laak A, Krause G, Heinrich N, Kühne R (2011). Chemogenomic Analysis of G-Protein Coupled Receptors and Their Ligands Deciphers Locks and Keys Governing Diverse Aspects of Signalling.. PloS One.

[46] Finn RD, Mistry J, Tate J, Coggill P, Heger A (2009). The Pfam protein families database.. Nucleic Acids Res.

[47] Bateman A, Punta M, Coggill P, Eberhardt R, Tate J (2011). Pfam version 24.. ftp://ftp.sanger.ac.uk/pub/databases/Pfam/releases/Pfam24.0/.

[48] Davies M, Al-Lazikani B, Bento AP, Gaulton A, Hersey A (2011). Kinase SARfari.. http://www.ebi.ac.uk/chembl/sarfari/kinasesarfari.

[49] R Development Core Team (2011). R: A Language and Environment for Statistical Computing. R Foundation for Statistical Computing, Vienna, Austria.. http://www.R-project.org.

[50] Vidal D, Thormann M, Pons M (2005). LINGO, an Efficient Holographic Text Based Method To Calculate Biophysical Properties and Intermolecular Similarities.. J Chem Inf Mod.

[51] OpenEye (2010). OEChem: version 1.7.4.. http://www.eyesopen.com.

