# Supplementary material for: Global Analysis of Small Molecule Binding to Related Protein Targets
Source: PLoS Comput Biol. 2012 Jan 12;8(1):e1002333. doi: 10.1371/journal.pcbi.1002333 (PMC3257267; doi:10.1371/journal.pcbi.1002333)
Supplement: Text S1 — This document provides additional data and analysis in support of the main text. In the first section, results of the mapping of small molecule binding to structural domains is discussed. The second and third section describe the species specific pharmacology of the histamine H3 receptor and the serotonin transporter. (PDF) [file pcbi.1002333.s017.pdf]

Global analysis of small molecule binding to related protein targets - Supporting Information

Felix A Kruger[1], John P Overington[1,*]
1 European Bioinformatics Institute, Hinxton, United Kingdom
∗ E-mail: jpo@ebi.ac.uk

Abstract

This document provides additional data and analysis in support of the main text. In the first section, results of the mapping of small molecule binding to structural domains is discussed. The second and third section describe the species specific pharmacology of the histamine H3 receptor and the serotonin transporter.

1 Supporting Information

1.1 A simple algorithm to predict binding site containing domains.

Protein domains are the evolutionary units that determine the structure and function of a protein. Many proteins consist of more than one domain and variation of the domain composition allows for functional diversification as seen for example in the role of SH2/SH3 domains in signaling pathways [1]. Binding sites for ligands to their targets in our analysis needed to be mapped to structural domains. Pfam [2] domains were retrieved for each target sequence and subsequently the domain that is most likely to contain the binding site was identified for each target, using a simple heuristic (see methods). According to this mapping most ChEMBL targets bind ligands through a small number of highly prevalent domains, while the vast majority of Pfam domains play no role in ligand binding (drug discovery). The occurrence of binding site containing domains in target proteins follows a power-law distribution [3], as illustrated in Figure S2. Validation of our method was therefore attempted by manually inspecting the prevalent domains and we found that mappings were plausible. A summary selection of the 25 most frequent domains - which account for more than 50% of all observations in the ChEMBL target dictionary - is shown in Table 2 in the main text. The most frequent domains are the 7tm_1 domain, which corresponds to the transmembrane domain of Class A GPCRs [4] and the Pkinase and Pkinase_Tyr domains, corresponding to Protein kinase and Tyrosine kinase sub-family domains. Beyond application in this current analysis, the mapping of binding sites to Pfam domains has the potential to improve the quality of annotation of pathway data.

1.2 Exploring the magic residue hypothesis: The Histamine H3 receptor.

A homology model of the Histamine H3 receptor (HRH3) was constructed from template structures of the Histamine H1 receptor (3rze), as well as the dopamine D3 receptor (3pbl), the human beta 2 adrenergic (2rh1, 3d4s, 3ny8, 3nya) and the turkey beta 1 adrenergic receptor (2vt4). PDB files were downloaded from www.pdbe.org and residues belonging to the chimeric T4 phage lysozyme part of the structure removed manually. PDB files of the human and rat homology model are deposited as supporting information datasets 1 and 2. The software used was MODELLER [5] and default parameters were chosen for the modeling. Inspection of the sequence revealed that Thr119 (which is substituted by Ala119 in the rat ortholog) in the human structure is aligned and spaced in a way that would allow H-bond formation with potential ligands (see Figure S3, S4). It is therefore tempting to speculate that the Thr119Ala substitution is the molecular basis of the species specific pharmacology of HRH3. In order to elucidate the role of the ligand chemotype in the sensing of crucial mutations in or near the binding site, we performed a basic clustering of the ligands tested against the HRH3. LINGO fingerprints have been previously

described as a fairly reliable fingerprint descriptor that is easy to calculate [6]. We used the OpenEye [7] software to calculate LINGO fingerprints for all HRH3 ligands in the analysis and to calculate pairwise Tanimoto coefficients. A distance matrix was constructed and hierarchical clustering carried out using the single linkage method to calculate distances between the clusters. Clusters were then determined using a Tanimoto cut-off of 0.5, as shown in Figure S5. This value was chosen arbitrarily with the aim to partition the ligand set into groups that could be more easily examined for a common chemotype. Thus we found that pyrrolidine containing antagonists (contained in cluster 24), bind the human HRH3 with higher affinity compared to the rat ortholog. Most imidazole containing HRH3 antagonists (contained in cluster 10) were found to have a higher affinity for the rat ortholog and indole containing (cluster 17) antagonists showed no preference for either ortholog of the human-rat pair (see Figure S6).

1.3 Exploring the magic residue hypothesis: The Serotonin transporter.

In analogy to the analysis performed on the Histamin H3 receptor, we probed the role of the ligand chemotype in the sensing of crucial mutations in or near the binding site for the Serotonin transporter. As for the HRH3, we performed a basic clustering of the ligands tested against the Serotonin receptor. For each ligand, a LINGO fingerprint was generated and pairwise distances calculated. Clusters were then determined using a Tanimoto cut-off of 0.5, as shown in Figure S7. We then examined the two biggest clusters, containing 121 and 42 ligands respectively. Compounds in the latter are characterized by an aminochroman-5-carboxamide core and the distribution of binding differences shows that the majority of these compounds has about ten-fold higher potency against the rat ortholog (see Figure S8). The larger cluster as well as all remaining compounds appear to have a slight preference for the rat ortholog.

References

1. Harrison SC (2003) Variation on an Src-like theme. Cell 112: 737–740.

2. Bateman A (2004) The Pfam protein families database. Nucl Acids Res 32: 138D–141.

3. Newman M (2005) Power laws, Pareto distributions and Zipf's law. Contemp Phys .

4. van der Horst E, Peironcely JE, Ijzerman AP, Beukers MW, Lane JR, et al. (2010) A novel chemogenomics analysis of G protein-coupled receptors (GPCRs) and their ligands: a potential strategy for receptor de-orphanization. BMC Bioinf 11: 316.

5. Šali A, Potterton L, Yuan F (1995) Evaluation of comparative protein modeling by MODELLER. Proteins 23: 318-326.

6. Vidal D, Thormann M, Pons M (2005) LINGO, an Efficient Holographic Text Based Method To Calculate Biophysical Properties and Intermolecular Similarities. J Chem Inf Mod 45: 386–393.

7. OpenEye (2010) OEChem: version 1.7.4. OpenEye Scientific Software Inc.Sante Fe. URL www.eyesopen.com.

Figure Legends

Figure 1. Histogram of the number of unique Pfam domains occuring in each protein in the ChEMBL target dictionary.

Figure 2. Cumulative log-log plot showing on the x-Axis the frequency of predicted binding-site containing domains among target proteins and on the y-Axis the number of domains with frequency >x. Over a large range of values the distribution can be approximated by a straight line, which is indicative of a power-law distribution. The fitted curve corresponds to a power-law function $p(x) = Cx^{-\alpha}$ where C is the number of domains (531) and α is 2.12.

Figure 3. Model structure of the human Histamine H3 recepor. Thr119 (which is substituted to Ala119 in the rat ortholog) is represented by a ball and stick model of the threonine side chain in the human receptor. Displayed ligands were adopted from the template structures.

Figure 4. Model structure of the human Histamine H3 recepor. Close-up view of the THR119 residue and measured distance to one of the template ligands, Doxepin (2.69A).

Figure 5. Hierachical clustering of HRH3 ligands. Cluster 24 is by far the largest cluster representing mainly pyrrolidine-containing antagonists while cluster 10 represents imidazole-containing antagonists.

Figure 6. Cluster specific distributions of differences in binding affinity for the HRH3. Most of the indole based antagonists of cluster 17 bind the rat ortholog with equal affinity, while the pyrrolidine-containing antagonists (contained in cluster 24) have a marked preference for the human receptor.

Figure 7. Hierachical clustering of Serotonin transporter ligands. The two largest clusters contain 121 and 42 compounds respectively.

Figure 8. (a) Cluster specific distributions of differences in binding affinity for the Serotonin transporter. The majority of compounds in cluster 22 have ten-fold higher potency against the rat ortholog. Cluster 26 and the majority of the remaining compounds (singletons) have a slight preference for the rat ortholog. (b) 2D representation of an exemplary compound from cluster 22.

Figure 9. Molecular weight and absolute differences in binding affinity. Box plots show distributions of differences in binding affinity for small molecules grouped by equally sized molecular weight bins for orthologs. Each bin contains the same number of values and lower bin limits are shown below each box. Anova type multiple testing was carried out to assess the significance of differences between neighbouring groups and levels of significance are indicated with one ($p < 5 * 10^{-2}$), two ($p < 5 * 10^{-5}$) or three asterisks ($p < 5 * 10^{-10}$). For orthologs, the only significant difference is between the group of compounds with molecular weight 375.5 - 422.6 Da and the group of compounds with molecular weight >483.3 Da.
